# Supplementary material for: The pRb/RBL2-E2F1/4-GCN5 axis regulates cancer stem cell formation and G0 phase entry/exit by paracrine mechanisms
Source: Nat Commun. 2024 Apr 27;15:3580. doi: 10.1038/s41467-024-47680-z (PMC11055877; doi:10.1038/s41467-024-47680-z)
Supplement: Supplementary file 1 — Supplementary Information [file 41467_2024_47680_MOESM1_ESM.pdf]

SUPPLEMENTARY INFORMATION

The pRb/RBL2-E2F1/4-GCN5 axis regulates cancer stem cell formation and G0 phase entry/exit by non-cell-autonomous mechanisms

Supplementary Table 1. Antibodies.

Antibody name	Techniques	Catalogue name	Company
Mouse anti-human Oct4	IF	sc-5279	Santa Cruz Biotechnology
Goat anti-human SOX2	IF	AF2018	R&D Systems
pRb mouse monoclonal	IF, WB	554136 (G3-245)	BD Pharmingen
RBL1/p107 (C-18) rabbit polyclonal	IF, WB	sc-318	Santa Cruz Biotechnology
RBL2/p130 (C-20) rabbit polyclonal	IF, WB, CHIP	sc-317	Santa Cruz Biotechnology
Actin mouse monoclonal	WB	MAB1501	Chemicon
E2F1 (C-20)	WB, CHIP	sc-193	Santa Cruz Biotechnology
E2F4 (A-20)	WB, CHIP	sc-1082x	Santa Cruz Biotechnology
WNT4 (m-70) rabbit polyclonal	WB	sc-13962	Santa Cruz Biotechnology
WNT5A (H-58) rabbit polyclonal	WB	sc-30224	Santa Cruz Biotechnology
WNT8A rabbit polyclonal	WB	SAB1411397	Sigma
WNT7B goat polyclonal	WB	AF3460	R&D Systems
P-ser33-B-cat rabbit polyclonal	WB	sc-16743-R	Santa Cruz Biotechnology
B-catenin (H-102) rabbit polyclonal	WB, CHIP	sc-7199	Santa Cruz Biotechnology
Alexa Fluor 568 donkey anti-mouse IgG	FACS	A-10037	Life Technologies
Alexa Fluor 568 donkey anti-rabbit IgG	FACS	A-10042	Life Technologies
Alexa Fluor 488 donkey anti-goat IgG	FACS	A-11055	Life Technologies
Alexa Fluor 568 donkey anti-goat IgG	FACS	A-11057	Life Technologies
Alexa Fluor 488 donkey anti-mouse	FACS	A-21202	Life Technologies
Alexa Fluor 488 donkey anti-rabbit IgG	FACS	A-21206	Life Technologies
Alexa Fluor 647 donkey anti-goat IgG	FACS	A-21447	Life Technologies
Alexa Fluor 647 donkey anti-mouse	FACS	A-31571	Life Technologies
Alexa Fluor 647 donkey anti-rabbit IgG	FACS	A-31573	Life Technologies

Supplementary Table 2. Primers used in Q-PCR.

Primer name	Primer Sequence
pRB	F ccaggccccctacctttgtcacc<br>R ttgttggtgttggcagaccttct
RBL1/p107	F ccaagaaagcgctctgctgtacaa<br>R acagacgcgtttggcagggg
RBL2/p130	F cccctctgatggagggacgcc<br>R ttggctgtgacagtggcggt
PBGD	F ggagccatgtctggtaacgg<br>R ccacgcgaatcactctcatct
Nanog	F catgagtgtggatccagcttg<br>R cctgaataagcagatccatgg
OCT4	F agtgagaggcaacctggaga<br>R acactcggaccacatccttc
SOX2	F tggacagttacgcgcacat<br>R cgagtaggacatgctgtaggt
WNT4	F cgtcttcggcaaggtggtga<br>R ctgaccccatgcactgtcct
WNT5A	F tgtgccacttgtatcaggacc<br>R tgcctatctgcatcaccctg
WNT8A	F ctgtggctgtgatgggtcaa<br>R ttcatcagggctctggcatc
WNT3	F ttgttccaactattgggggc<br>R gctgtgagcccagagatgtg

Supplementary Table 3. Primers used in Chromatin Immunoprecipitation.

Primer name	Primer Sequence
WNT4	F gccaagagagcttccctaaact<br>R atccgaaacctcgcttctgg
WNT3	F tgaacccctcaaggaggaga<br>R acggagccgagtgtcatttg
WNT7B	F caatctgacttcgggctctc<br>R gatgacagacgggctaacct
WNT8A	F ctgggtggccctaaggttg<br>R ccatctctgcaacagtccct
WNT2B for E2F1 ChIP Pos.1	F ggaagccatgtggttctgac<br>R ctgtccttccctcagagaggt
WNT2B for E2F1 ChIP Pos.2	F cccgctcctaaggttttctt<br>R cgctgagctttagccaagat
WNT2B for E2F1 ChIP Pos.3	F ttgctctggtttcacatcca<br>R ccctccttaatcccaccatt
WNT9A for E2F1 ChIP Pos.1	F acaagcccagggtgtgtc<br>R ccatctgtgcaacccgtag
WNT3A for E2F1 ChIP Pos.1	F cagccagagggatatctggt<br>R tgattcaccggatgaggaat
WNT3A for E2F1 ChIP Pos.2	F gaaggttccatgaagcgagt<br>R taagcttggtcttggggttg
WNT10A for E2F1 ChIP	F agaagcaggccaggctaag<br>R cacctgccagcacgtctt
WNT7A for E2F1 ChIP	F agggactgggaggtgacttt<br>R ccccttcatgagagtgtggt
WNT2 for E2F1 ChIP	F aagctcagagacgccaaaaa<br>R ccctgctcttcccgagtc
WNT11 for E2F1 ChIP	F gtcaccttccgcctgagc<br>R atccccagacggagaagc
WNT5B for E2F1 ChIP Pos.1	F gagctgggagtagggtaggg<br>R actcacacgcacacttacgc
WNT5B for E2F1 ChIP Pos.2	F gtggcccctgatttttaacc<br>R attgcaggggtaaactgtgg
WNT5B for E2F1 ChIP Pos.3	F caaaagacaagggggaatca<br>R tccagataggactttccagca
WNT5B for E2F1 ChIP Pos.4	F atgtcacatgatgccactgc<br>R caggcaagctgttccaact
WNT5B for E2F1 ChIP Pos.5	F ccaaaggatcagaggagcag<br>R cggtcagactaacccgtttc
WNT10B for E2F1 ChIP Pos.1	F aaaggactgggggctctg<br>R gtcaaggctgggagtggag
WNT7B for E2F1 ChIP Pos.1	F ggttgaaccagagccaagaa<br>R ggttccccgtgagacagtaa
WNT7B for E2F1 ChIP Pos.2	F ccatccgcaaataaagccta<br>R accattgctgttgctcgaat
WNT7B for E2F1 ChIP Pos.3	F aaccctctggtctctcgtca<br>R aaaccagacaacacaggcaac
WNT7B for E2F1 ChIP Pos.4	F acagcacttctgggactgtg<br>R caagtgagtcatgggtgacg

Supplementary Table 4. Human primer sequences used in luciferase assay.

Gene name	Primer Sequence
WNT4 F	TCTTACGCGTGCTAGTATGGGAGGATAAATCGAACAAGCACATAGAA AACCAAATGCAAA
WNT4 R	TCTCGAGCTAGCGGCGGCGGAGGCGGGCG
WNT8A F	TCTTACGCGTGTAAATAAATAAATAAATAAATAAATAAATAAAATAAAATAAAAGACAGATTCC CACCTAAGGAGCTGAAGTCCTAGTAA
WNT8A R	TCTCGAGCTAGATTTCTTTCTTCTATATGTCAATTTTGTCTCTTCCTGCTTCTTTGGCCC

Supplementary Table 5. Antibodies used for the detection of GFP-OCT4, CD133 and SSEA4 by flow cytometry in the compound screening experiment.

Antibody	Target/function	Ratio	Number of cells
CD133-BV786, Mouse Anti-Human, clone W6B3C1, BD 747640	CD133	0.6 uL in 100 uL	300,000
Mouse IgG1-BV786, BD 563330	isotype control for CD133	0.6 uL in 100 uL	300,000
Alexa647 Mouse anti-SSEA-4 clone MC813-70	SSEA4	2.5 uL to 100 uL	300,000
Alexa647 Mouse IgG3	isotype control for SSEA4	0.31 uL to 100 uL	300,000

Supplementary Table 6. Fold changes and statistical significance of each genes in PDAC_C1 cluster compared to all other PDAC cancer cell clusters C2-C8 combined.

Gene Symbol	P values	log2(average fold change)	Adjusted P value
WNT7B	1.7E-12	0.500911692	6.13E-08
WNT7A	1.1E-06	0.103666514	4.05E-02
WNT10A	2.6E-14	0.491873821	9.53E-10
WNT4	3.7E-36	0.606578146	1.34E-31
CCND1	5.1E-90	1.055247741	1.86E-85
CCNE1	5.4E-01	0.049239927	1.00E+00
FZD6	2.0E-24	0.347205477	7.42E-20
FZD5	1.6E-54	1.378857695	5.93E-50
E2F1	5.2E-02	0.258231879	1.00E+00
E2F4	2.0E-19	0.093530481	7.17E-15
CDK4	1.6E-03	0.076765712	1.00E+00
CDK6	1.8E-21	0.110738306	6.69E-17
CDK2	4.5E-05	0.007054789	1.00E+00
ALDH1A1	1.7E-72	1.574436323	6.28E-68
PROM1	1.5E-43	1.404276581	5.56E-39
KLF4	1.5E-36	0.501374677	5.33E-32

Supplementary Table 7. Top 20 transcription factor motifs found in ATAC-seq peaks for A13A non-CSCs.

Total Target Sequences = 52059, Total Background Sequences = 49524

Rank	Motif	Name	P-value	log P-pvalue	q-value (Benjamini)	# Target Sequences with Motif	% of Targets Sequences with Motif	# Background Sequences with Motif	% of Background Sequences with Motif
1	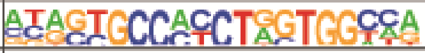	CTCF(Zf)/CD4+-CTCF-ChIP-Seq(Barski_et_al.)/Homer	1e-4598	-1.059e+04	0.0000	9683.0	18.60%	1426.3	2.88%
2	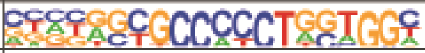	BORIS(Zf)/K562-CTCFL-ChIP-Seq(GSE32465)/Homer	1e-3151	-7.256e+03	0.0000	11910.0	22.88%	3221.6	6.50%
3	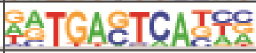	Fosl2(bZIP)/3T3L1-Fosl2-ChIP-Seq(GSE56872)/Homer	1e-1538	-3.542e+03	0.0000	7847.0	15.08%	2553.8	5.16%
4	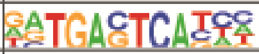	Jun-AP1(bZIP)/K562-cJun-ChIP-Seq(GSE31477)/Homer	1e-1477	-3.402e+03	0.0000	6467.0	12.42%	1875.8	3.79%
5	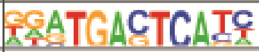	Fra2(bZIP)/Striatum-Fra2-ChIP-Seq(GSE43429)/Homer	1e-1432	-3.299e+03	0.0000	9677.0	18.59%	3762.9	7.60%
6	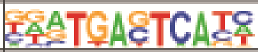	Fra1(bZIP)/BT549-Fra1-ChIP-Seq(GSE46166)/Homer	1e-1400	-3.225e+03	0.0000	10617.0	20.40%	4405.7	8.90%
7	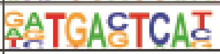	JunB(bZIP)/DendriticCells-Junb-ChIP-Seq(GSE36099)/Homer	1e-1356	-3.124e+03	0.0000	10486.0	20.15%	4388.1	8.86%
8	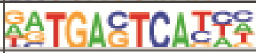	Atf3(bZIP)/GBM-ATF3-ChIP-Seq(GSE33912)/Homer	1e-1234	-2.843e+03	0.0000	11633.0	22.35%	5363.2	10.83%
9	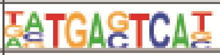	BATF(bZIP)/Th17-BATF-ChIP-Seq(GSE39756)/Homer	1e-1219	-2.808e+03	0.0000	11386.0	21.88%	5222.0	10.54%
10	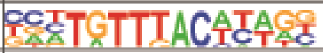	Foxa3(Forkhead)/Liver-Foxa3-ChIP-Seq(GSE77670)/Homer	1e-1187	-2.734e+03	0.0000	7736.0	14.86%	2912.1	5.88%
11	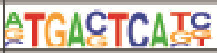	AP-1(bZIP)/ThioMac-PU.1-ChIP-Seq(GSE21512)/Homer	1e-1142	-2.631e+03	0.0000	12335.0	23.70%	6033.1	12.18%
12	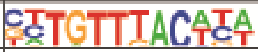	Foxa2(Forkhead)/Liver-Foxa2-ChIP-Seq(GSE25694)/Homer	1e-1090	-2.511e+03	0.0000	13872.0	26.65%	7265.4	14.67%
13	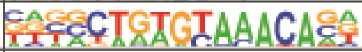	Fox:Ebox(Forkhead,bHLH)/Panc1-Foxa2-ChIP-Seq(GSE47459)/Homer	1e-1025	-2.361e+03	0.0000	16159.0	31.05%	9174.6	18.52%
14	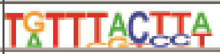	FOXM1(Forkhead)/MCF7-FOXM1-ChIP-Seq(GSE72977)/Homer	1e-962	-2.217e+03	0.0000	16134.0	31.00%	9323.1	18.82%
15	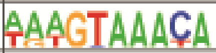	FOXA1(Forkhead)/MCF7-FOXA1-ChIP-Seq(GSE26831)/Homer	1e-873	-2.011e+03	0.0000	15782.0	30.32%	9296.1	18.77%
16	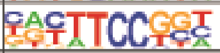	Fli1(ETS)/CD8-FLI-ChIP-Seq(GSE20898)/Homer	1e-870	-2.005e+03	0.0000	18764.0	36.05%	11728.2	23.68%
17	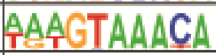	FOXA1(Forkhead)/LNCAP-FOXA1-ChIP-Seq(GSE27824)/Homer	1e-834	-1.921e+03	0.0000	17734.0	34.07%	10993.6	22.20%
18	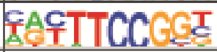	Elk4(ETS)/Hela-Elk4-ChIP-Seq(GSE31477)/Homer	1e-809	-1.864e+03	0.0000	11711.0	22.50%	6328.3	12.78%
19	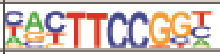	Elk1(ETS)/Hela-Elk1-ChIP-Seq(GSE31477)/Homer	1e-776	-1.788e+03	0.0000	11802.0	22.67%	6481.5	13.09%
20	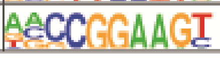	ELF1(ETS)/Jurkat-ELF1-ChIP-Seq(SRA014231)/Homer	1e-693	-1.596e+03	0.0000	10983.0	21.10%	6082.6	12.28%

Supplementary Table 8. Top 20 transcription factor motifs found in ATAC-seq peaks for A13A CSCs.

Total Target Sequences = 64398, Total Background Sequences = 61951

Rank	Motif	Name	P-value	log P-value	q-value (Benjamini)	#Target Sequences with Motif	% of Target Sequences with Motif	# Background Sequences with Motif	% of Background Sequences with Motif
1	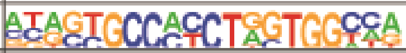	CTCF(Zf)/CD4+-CTCF-ChIP-Seq(Barski_et_al.)/Homer	1e-4208	-9.690e+03	0.0000	9840.0	15.28%	1655.5	2.64%
2	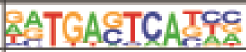	Fosl2(bZIP)/3T3L1-Fosl2-ChIP-Seq(GSE56872)/Homer	1e-3404	-7.840e+03	0.0000	12716.0	19.74%	3395.5	5.48%
3	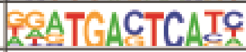	Fra2(bZIP)/Striatum-Fra2-ChIP-Seq(GSE43429)/Homer	1e-3260	-7.507e+03	0.0000	15558.0	24.15%	5067.6	8.19%
4	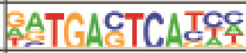	Jun-AP1(bZIP)/K562-cJun-ChIP-Seq(GSE31477)/Homer	1e-3258	-7.502e+03	0.0000	10525.0	16.34%	2447.2	3.95%
5	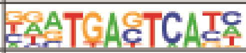	Fra1(bZIP)/BT549-Fra1-ChIP-Seq(GSE46166)/Homer	1e-3159	-7.275e+03	0.0000	17001.0	26.39%	6034.8	9.75%
6	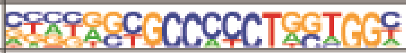	BORIS(Zf)/K562-CTCFL-ChIP-Seq(GSE32465)/Homer	1e-3120	-7.185e+03	0.0000	12146.0	18.86%	3339.6	5.38%
7	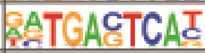	JunB(bZIP)/DendriticCells-Junb-ChIP-Seq(GSE36099)/Homer	1e-3017	-6.947e+03	0.0000	16693.0	25.91%	6014.2	9.71%
8	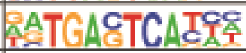	Atf3(bZIP)/GBM-ATF3-ChIP-Seq(GSE33912)/Homer	1e-2854	-6.572e+03	0.0000	18374.0	28.52%	7288.9	11.77%
9	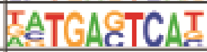	BATF(bZIP)/Th17-BATF-ChIP-Seq(GSE39756)/Homer	1e-2744	-6.320e+03	0.0000	18012.0	27.96%	7203.2	11.63%
10	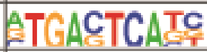	AP-1(bZIP)/ThioMac-PU.1-ChIP-Seq(GSE21512)/Homer	1e-2576	-5.932e+03	0.0000	19136.0	29.71%	8202.0	13.25%
11	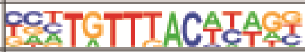	Foxa3(Forkhead)/Liver-Foxa3-ChIP-Seq(GSE77670)/Homer	1e-2127	-4.899e+03	0.0000	11507.0	17.86%	3965.9	6.41%
12	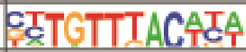	Foxa2(Forkhead)/Liver-Foxa2-ChIP-Seq(GSE25694)/Homer	1e-2034	-4.684e+03	0.0000	20323.0	31.55%	9984.8	16.13%
13	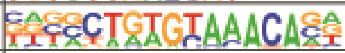	Fox:Ebox(Forkhead,bHLH)/Panc1-Foxa2-ChIP-Seq(GSE47459)/Homer	1e-2008	-4.625e+03	0.0000	23094.0	35.85%	12130.1	19.59%
14	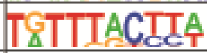	FOXM1(Forkhead)/MCF7-FOXM1-ChIP-Seq(GSE72977)/Homer	1e-1793	-4.130e+03	0.0000	23692.0	36.78%	13081.5	21.13%
15	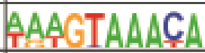	FOXA1(Forkhead)/MCF7-FOXA1-ChIP-Seq(GSE26831)/Homer	1e-1735	-3.996e+03	0.0000	23354.0	36.25%	12951.8	20.92%
16	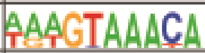	FOXA1(Forkhead)/LNCAP-FOXA1-ChIP-Seq(GSE27824)/Homer	1e-1656	-3.815e+03	0.0000	26053.0	40.44%	15317.0	24.77%
17	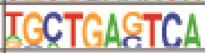	Bach2(bZIP)/OCILy7-Bach2-ChIP-Seq(GSE44420)/Homer	1e-1408	-3.243e+03	0.0000	6580.0	10.21%	2002.0	3.23%
18	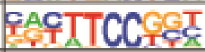	Fli1(ETS)/CD8-FLI-ChIP-Seq(GSE20898)/Homer	1e-960	-2.211e+03	0.0000	22156.0	34.40%	14154.5	22.86%
19	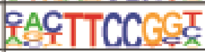	Elk1(ETS)/Hela-Elk1-ChIP-Seq(GSE31477)/Homer	1e-933	-2.148e+03	0.0000	13295.0	20.64%	7184.1	11.60%
20	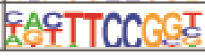	Elk4(ETS)/Hela-Elk4-ChIP-Seq(GSE31477)/Homer	1e-931	-2.145e+03	0.0000	13206.0	20.50%	7122.9	11.50%

SUPPLEMENTARY FIGURES

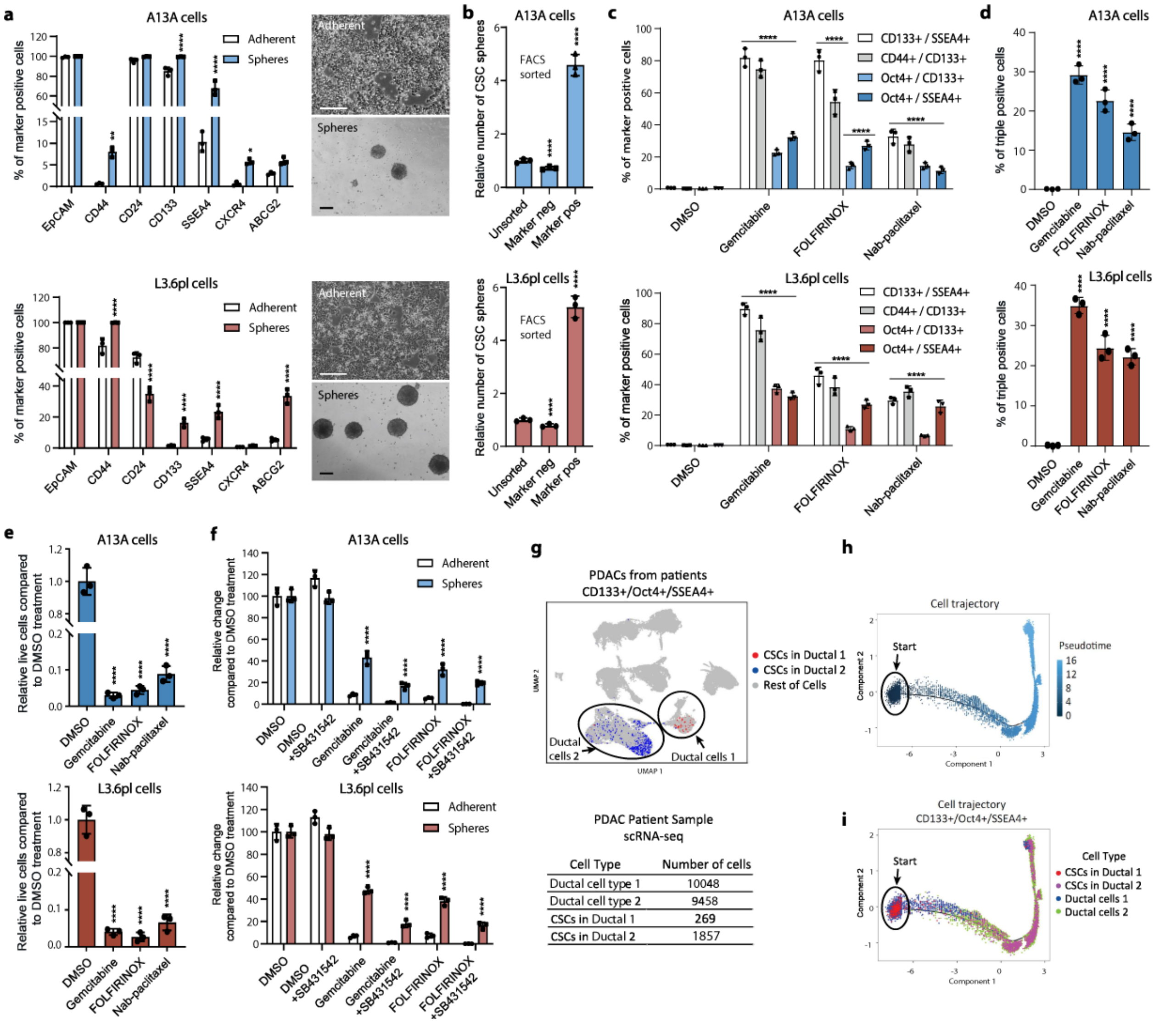

Supplementary Figure 1: Characterising CSCs in PDAC cell lines. (**a**) Characterisation of cancer stem cell marker expression in PDAC cells grown in adherent conditions that supports non-CSC proliferation, and CSC sphere condition which enriches for anoikis-resistant CSCs. A13A and L3.6pl cells were grown in adherent and as CSC spheres, followed by flow cytometry analysis of markers. A13A CSCs indicate the enrichment of CD44, CD133, SSEA4 and CXCR4 markers, whereas L3.6pl CSCs indicate the enrichment of CD44, CD133, SSEA4 and ABCG2 markers. Representative images of adherent cells spheres are shown. Scale bars 50µm. (**b**) Self-renewal of Oct4/CD133/SSEA4 CSC marker positive and negative cell populations in A13A and L3.6pl cells sorted by FACS followed by CSC sphere assays. (**c-e**) Chemotherapy reagents enrich for cells expressing Oct4+/CD133+/SSEA4+ CSC markers. A13A and L3.6pl cells were treated with 0.5µM Gemcitabine, 0.5µM FOLFIRINOX and 0.5µM Paclitaxel for 5 days and analysed by flow cytometry (c) for detecting CSC marker double positive cells, (d) OCT4-GFP+/CD133+/SSEA4+ triple positive cells, and (e) live cells upon each treatments. N=3 independent experiments in all graphs. Statistical analysis in b, d, e was performed by multiple t test. (**f**) TGFβ/Activin signalling induces chemoresistant OCT4-GFP+/CD133+/SSEA4+ CSCs. CSC spheres are more resistant to Gemcitabine, 5-FU and Paclitaxel treatment than adherent-cultured non-CSCs. A13A and L3.6pl cells were treated with 0.5µM Gemcitabine, 0.5µM FOLFIRINOX and 0.5µM Paclitaxel in combination with Activin A or 10 µM SB431542 for 5 days. N=3 independent experiments in all graphs. Statistical analysis in a, c, f was performed by 2-way ANOVA with multiple comparisons with Tukey correction and **** marks adjusted P-value <0.0001, *** is adjusted P-value <0.001, ** is adjusted P-value <0.01, * is adjusted P-value <0.05. (**g-i**) Developmental trajectory analysis of CSCs in PDAC patient tumours based on single-cell RNA-sequencing analysis of patient tumour samples. (g) UMAP of cell populations in patient tumours. Ductal 1 and ductal 2 cell are cancer cell populations (circled populations), which contain Oct4+/CD133+/EPCAM+ cancer cells marked as CSCs in Ductal 1 (red) and CSCs in Ductal 2 (blue) population. The rest of the cells contain various cell types found in patient tumours (grey). Cluster information is shown. The table below UMAP plot indicates the number of cells for each cell population, and CSC cell numbers mark Oct4+/CD133+/EPCAM+ cancer cells. (h) Developmental trajectory of PDAC ductal cells based on single-cell RNA-sequencing analysis of patient tumour samples. Pseudo-time 0 is the starting point. Pseudo-time of ductal cells was inferred by Monocle2 and each point corresponds to a single cell. (**i**) Developmental trajectory of Oct4+/CD133+/EPCAM+ CSCs. Oct4+/CD133+/EPCAM+ CSCs progress on the developmental trajectory indicating that CSCs (red) in Ductal 1 (blue) is the earlier stage that gives rise to CSCs (purple) in Ductal 2 cells (green). These populations depict the same cells described in (g). Data are presented as mean values +/- SD. Source data are provided as a Source Data file.

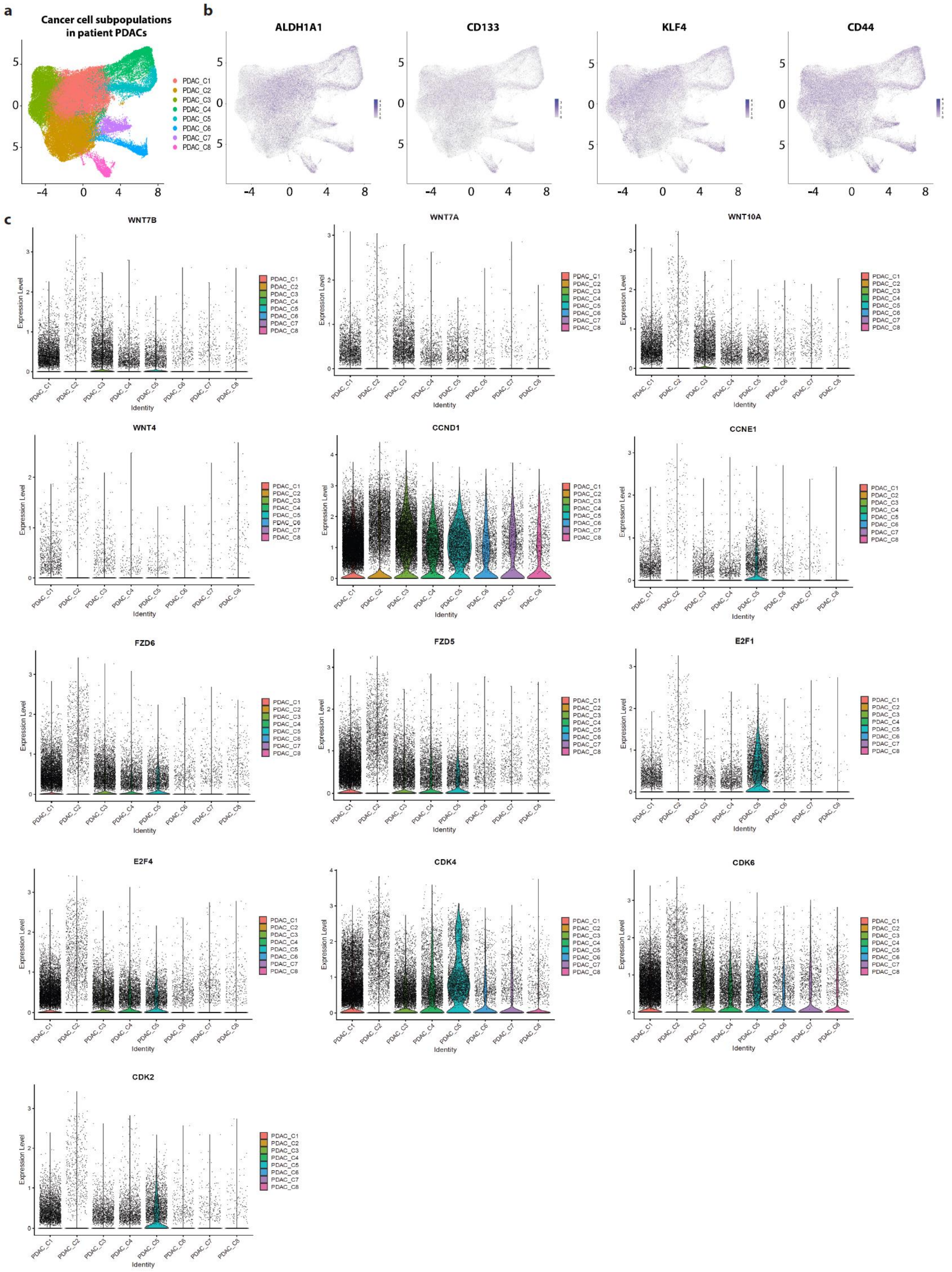

Supplementary Figure 2: Expression of WNT ligands in subpopulations of cancer cells in primary tumour samples analysed by single-cell RNA-sequencing. (**a**) Clustering of cancer cell subpopulations in PDAC patient tumour sample RNA-sequencing data. (**b**) Expression of CSC markers ALDH1A1, CD133, KLF4 and CD44 in cancer cell subpopulations in patient tumours. (**c**) WNT ligand expression in cancer cell subpopulations in patient tumours.

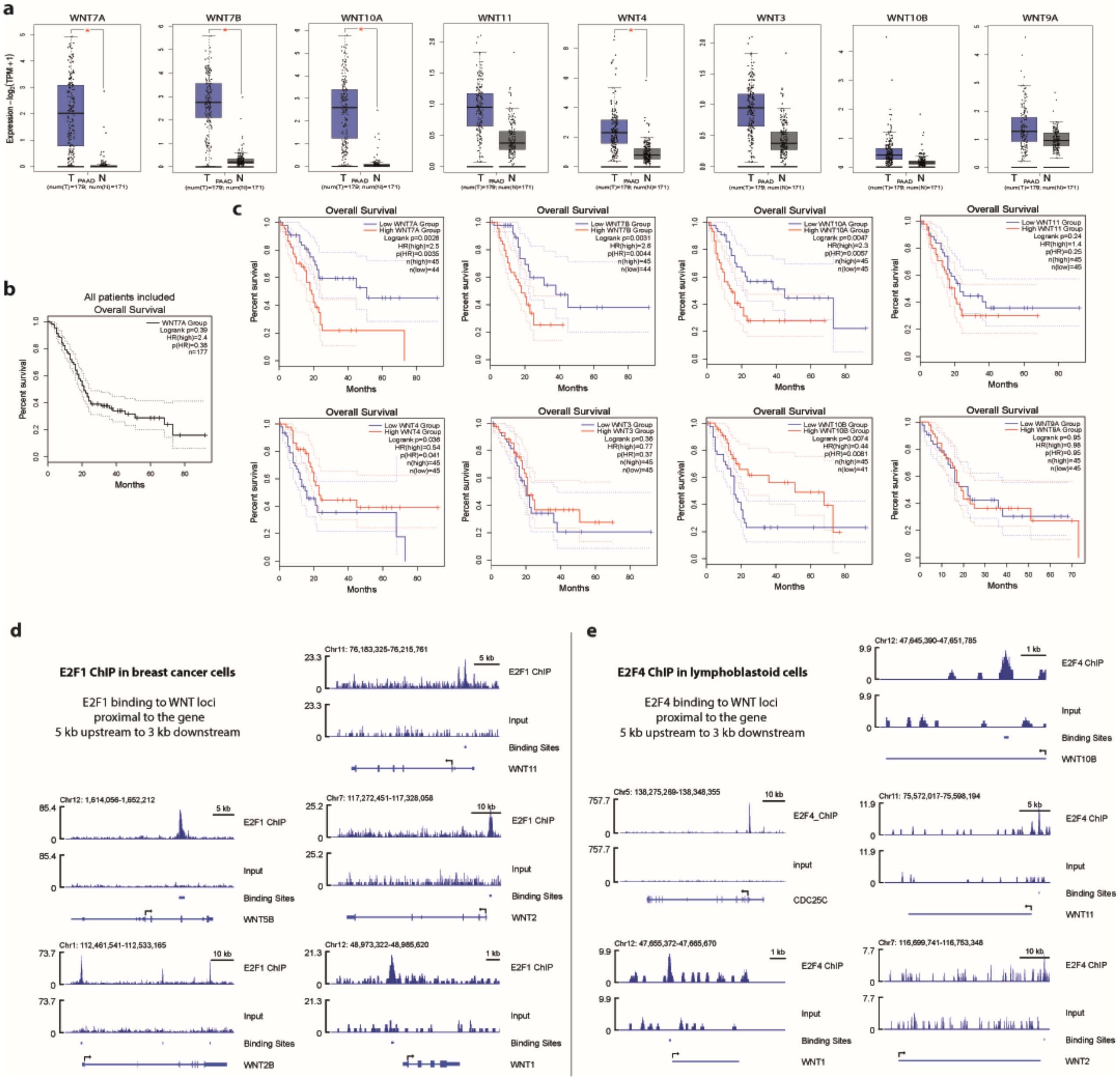

Supplementary Figure 3: E2F1 and E2F4 bind to WNT loci. (**a**) WNT7A, WNT7B and WNT10A have elevated expression in PDACs patient samples compared to normal pancreatic tissue. Statistical analysis is one-way ANOVA with p-value significance 0.01. The horizontal line shows mean value +/- SD. (**b**) Overall PDAC patient mortality. (**c**) Higher expression of WNT7A, WNT7B and WNT10A correlates with lower survival of pancreatic cancer patients. (**d-e**) E2F1 and E2F4 binding to WNT loci based on ChIP-seq data. (**d**) Genomic views of E2F1 ChIP-seq data in breast cancer cells showing E2F1 binding to WNT loci proximal to the gene 5 kb upstream to 3 kb downstream. (**e**) Genomic views of E2F4 ChIP-seq data in lymphoblastoid cells showing E2F1 binding to WNT loci proximal to the gene 5 kb upstream to 3 kb downstream.

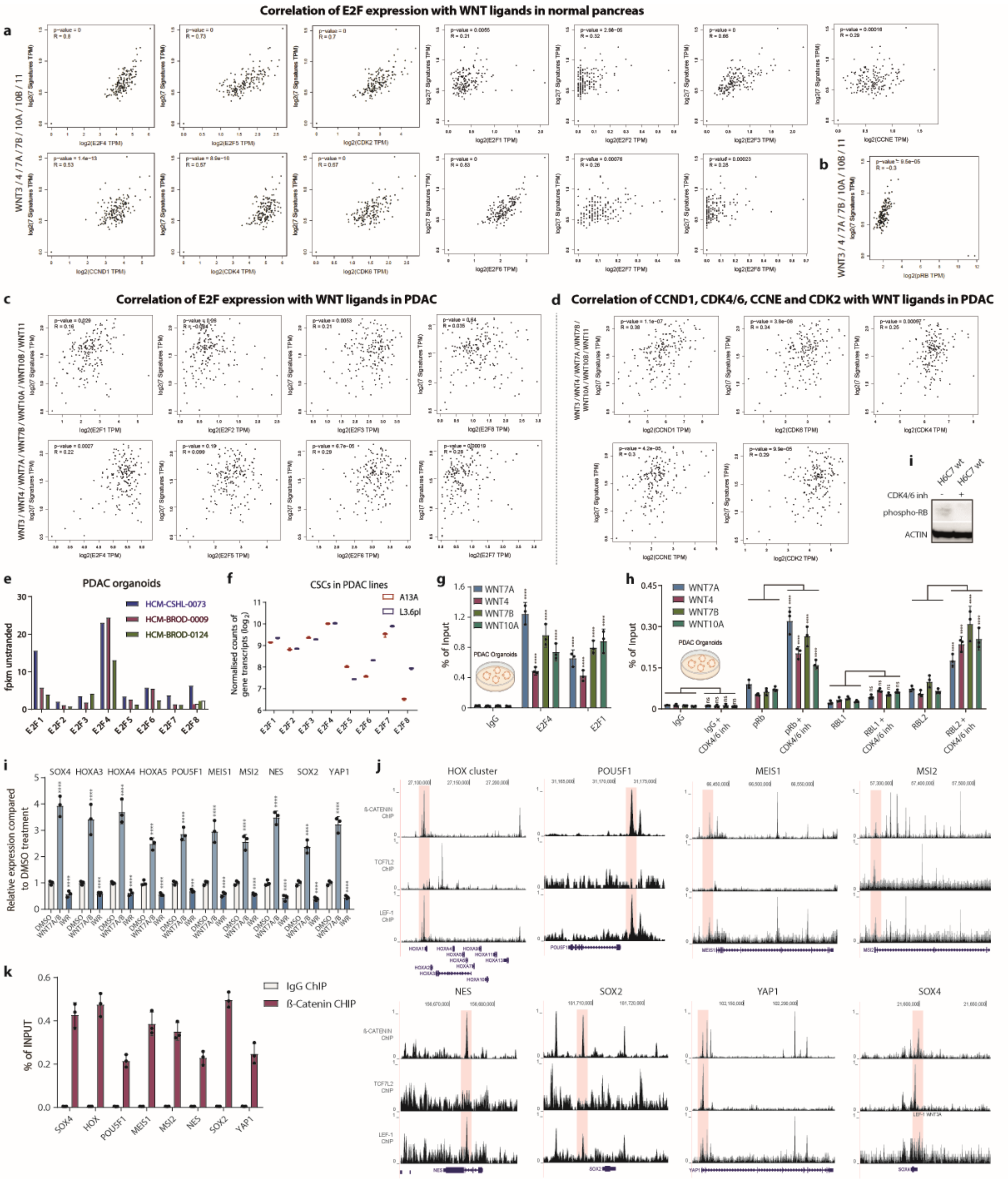

Supplementary Figure 4: WNT signalling stimulates β-catenin binding to CSC self-renewal loci and induces their expression in CSCs. (**a**) Correlation of E2F expression with WNT ligands in normal human pancreas. (**b**) WNT ligand expression is negatively correlated with pRb expression in normal pancreatic tissue. (**c**) Correlation of E2F expression with WNT ligands in human PDACs. (**d**) Correlation of CCND1, CDK4/6, CCNE and CDK2 with WNT ligands in PDAC. (**e**) Expression levels of each E2F family member in three PDAC patient-derived organoids based on RNA-sequencing data. (**f**) Normalised counts of E2F family gene transcripts (log2) in A13A and L3.6pl PDAC CSCs show the highest expression for E2F4 similar to PDAC patient-derived organoids. (**g**) E2F4 and E2F1 bind to the regulatory regions near WNT ligand loci in PDAC organoids. (**h**) CDK4/6 inhibition increases the binding of RB family proteins to WNT ligand loci in PDAC organoids. (**i**) WNT signalling induces the expression of CSC self-renewal factors in CSCs. (**j**) β-catenin binds to CSC self-renewal loci as shown by genomic views of β-catenin ChIP-seq data. (**k**) β-catenin binds to CSC self-renewal loci in pancreatic CSCs according to ChIP-qPCR data. Data are presented as mean values +/- SD. Statistical analysis in g, h, I, k was performed by 2-way ANOVA with multiple comparisons with Tukey correction and **** marks adjusted P-value <0.0001, *** is adjusted P-value <0.001, ** is adjusted P-value <0.01, * is adjusted P-value <0.05. N=3 independent experiments in all graphs. Source data are provided as a Source Data file.

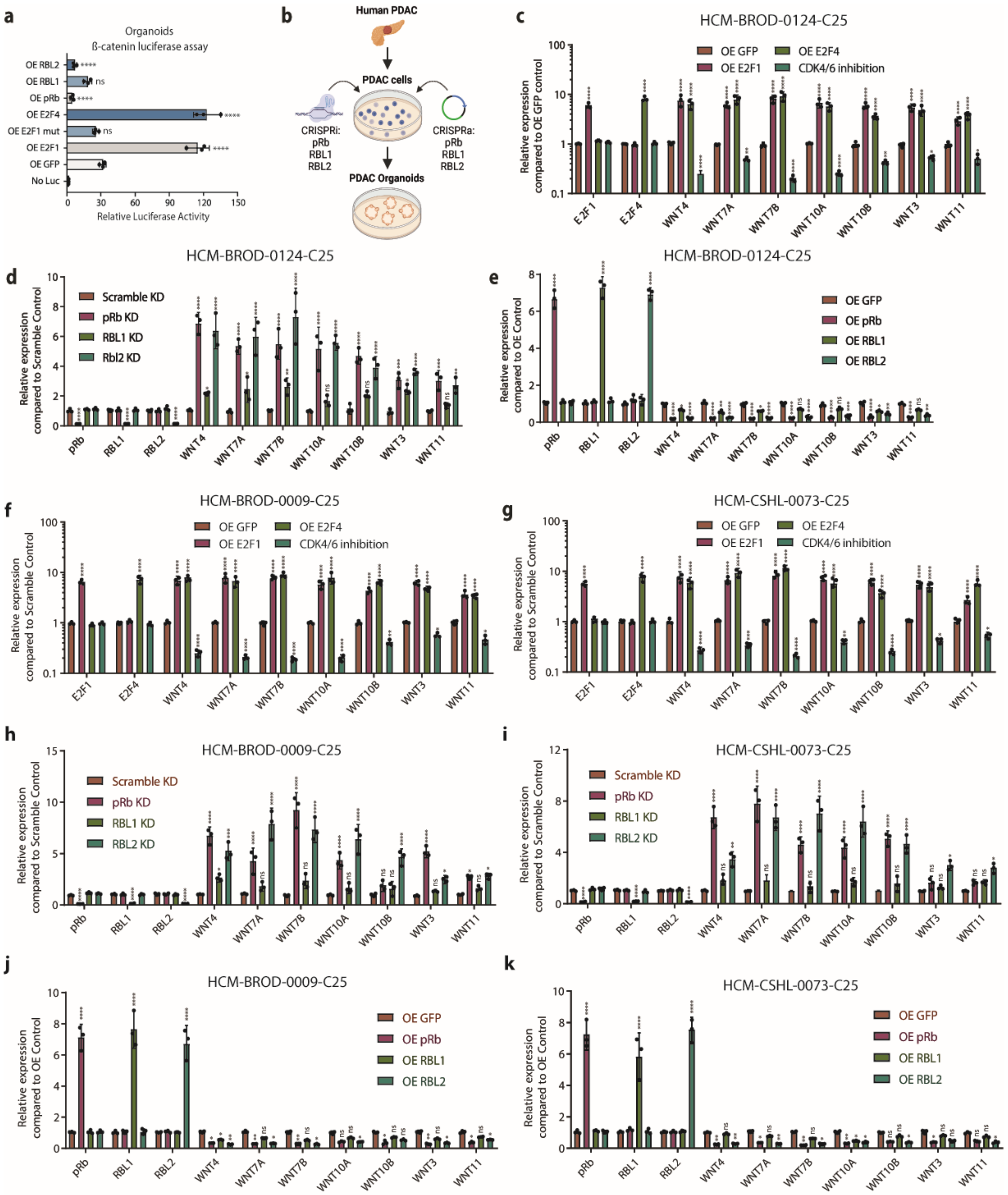

Supplementary Figure 5: E2F1 and E2F4 induce WNT ligand expression while pRb and RBL2 reduce WNT ligand expression in PDAC patient-derived organoids. (**a**) Promoter luciferase analysis with construct containing TCF/LEF sites upstream of a luciferase reporter (M50 Super 8x TOPFlash). N=3 independent experiments. Statistical analysis was performed by multiple t test. **** marks adjusted P-value <0.0001, *** is adjusted P-value <0.001, ** is adjusted P-value <0.01, * is adjusted P-value <0.05. (**b**) Schematic depiction of CRISPRi knockdown and CRISPRa induction of RB proteins in PDAC organoids. (**c**-**k**) Overexpression of E2F1 and E2F4 induce but CDK4/6 inhibition reduces WNT ligand expression, whereas knockdown of RBs induce WNT ligand expression in organoids. Data are presented as mean values +/- SD. Statistical analysis in c-k was performed by 2-way ANOVA with multiple comparisons with Tukey correction and **** marks adjusted P-value <0.0001, *** is adjusted P-value <0.001, ** is adjusted P-value <0.01, * is adjusted P-value <0.05. N=3 independent experiments in all graphs. Source data are provided as a Source Data file.

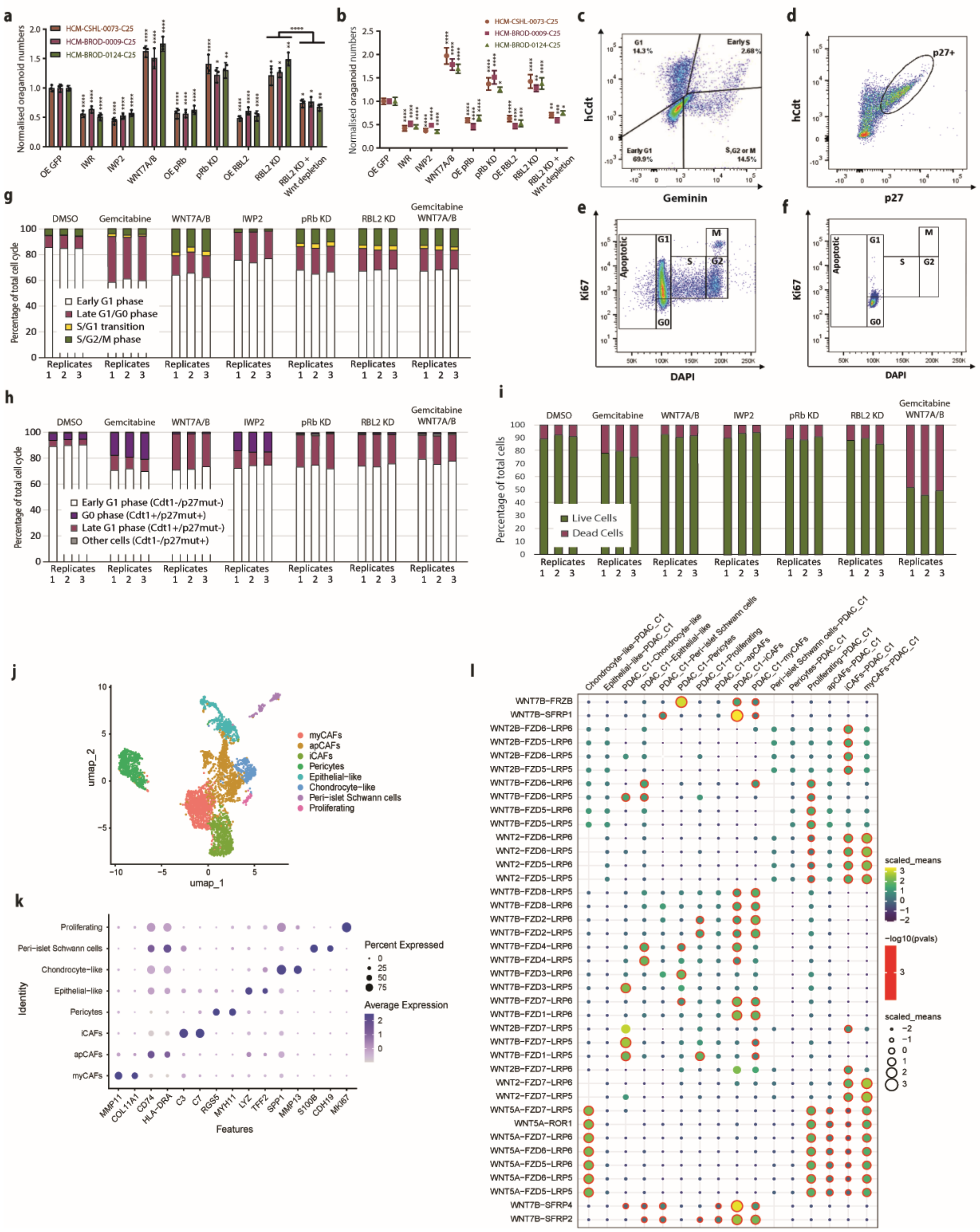

Supplementary Figure 6: Crosstalk between CSCs and different subtypes of cancer-associated fibroblasts. (**a-b**) Paracrine effects of RBs on organoids are mediated at least partly by WNT ligands. Conditioned media collected from RB OE or KD cells or WNT signalling impact (**a**) organoid numbers and (**b**) organoid sizes. Data are presented as mean values +/- SD. N=3 independent experiments. Statistical analysis was performed by 2-way ANOVA with multiple comparisons with Tukey correction and **** marks adjusted P-value <0.0001, *** is adjusted P-value <0.001, ** is adjusted P-value <0.01, * is adjusted P-value <0.05. (**c-d**) A13A-FUCCI cell characterisation with G0 phase detection by flow cytometry. (**g-h**) Non-cell-autonomous effects of pRb and RBL2 on cell cycle progression. Advanced three-colour FUCCI cells were treated with conditioned media collected from pRb KD or RBL2 KD and analysed for the percentages of cells in each cell cycle phase upon the specific treatments. (**i**) The ratio of live and dead cells measure by DAPI in each treatment condition. (**j**) UMAP of the different subtypes of fibroblasts in PDAC patient tumours. (**k**) Marker genes used to detect the different subtypes of fibroblasts in PDAC patient tumours. (**l**) Paracrine signalling between CSCs (PDAC Cluster 1) and subtypes of fibroblasts in PDAC patient primary tumours. Source data are provided as a Source Data file.

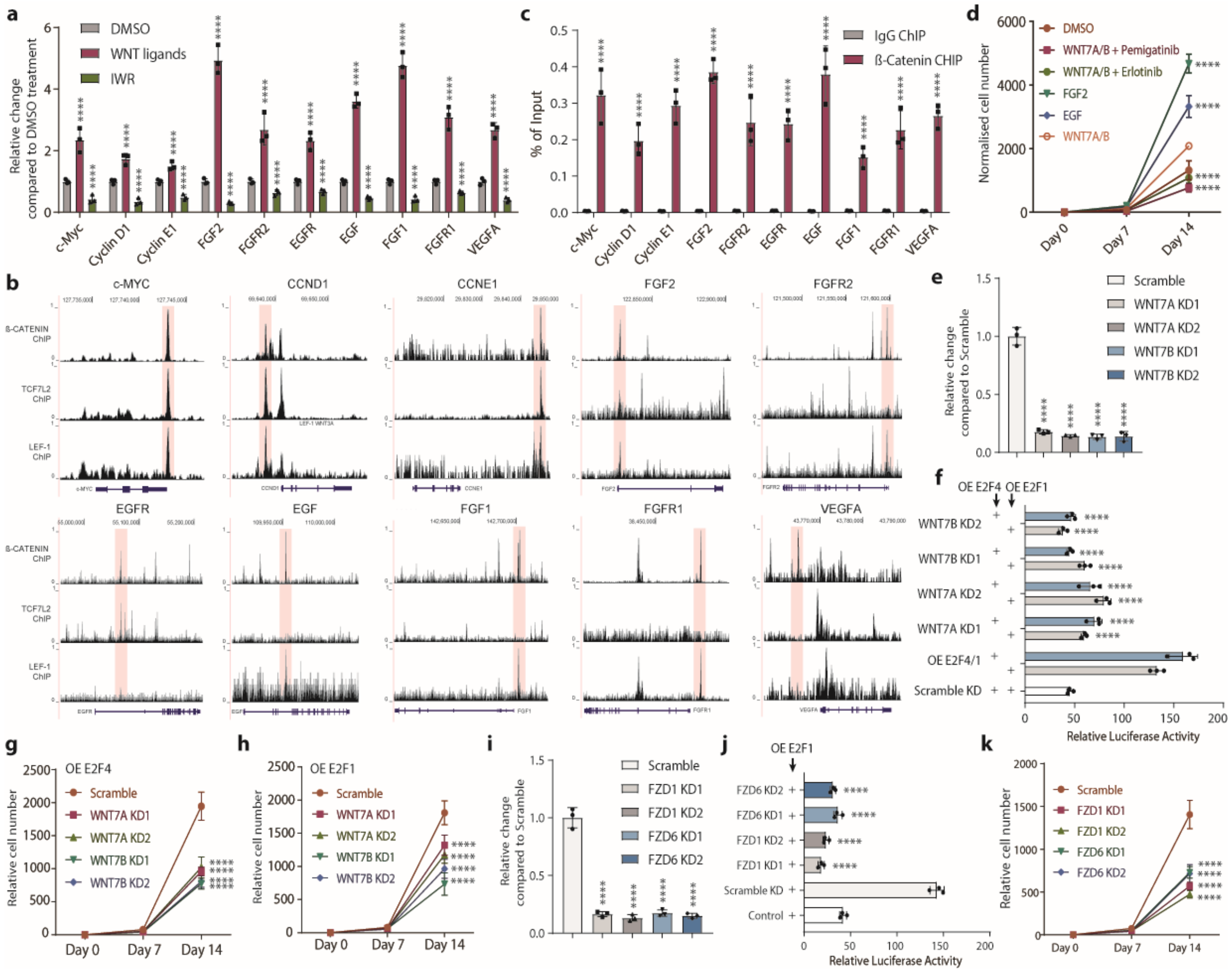

Supplementary Figure 7: Paracrine WNT-β-catenin signalling regulates CSCs and fibroblasts. (**a**) WNT signalling induces the expression of cell cycle regulatory factors in fibroblasts. (**b**) β-catenin binds to cell cycle regulatory loci as shown by genomic views of β-catenin ChIP-seq data. (**c**) β-catenin binds to cell cycle regulatory loci in pancreatic CSCs according to ChIP-qPCR data. (**d**) The impact of FGF and EGF pathway on fibroblast proliferation. (**e**) WNT7A and WNT7B KD clone confirmation by qPCR. (**f**) WNT7A/B KD in CSCs reduces β-catenin dependent promoter-luciferase signalling in fibroblasts in co-culture conditions thereby proving paracrine crosstalk between CSCs and fibroblasts. (**g-h**) WNT7A/B KD in CSCs that overexpress E2F4/1 reduces fibroblast proliferation. (**i**) FZD1 and FZD6 KD clone confirmation by qPCR. (**j**) FZD1 and FZD6 KD in fibroblast reduces β-catenin dependent promoter-luciferase signalling in fibroblasts in co-culture conditions thereby proving paracrine crosstalk between CSCs. Data are presented as mean values +/- SD. Statistical analysis in e, i, j was performed by multiple t test. **** marks adjusted P-value <0.0001, *** is adjusted P-value <0.001, ** is adjusted P-value <0.01, * is adjusted P-value <0.05. N=3 independent experiments in all graphs. (**k**) The impact of FZD1 and FZD6 KD reduces fibroblast proliferation. Statistical analysis in a, c, d, f, g, h, k was performed by 2-way ANOVA with multiple comparisons with Tukey correction and **** marks adjusted P-value <0.0001, *** is adjusted P-value <0.001, ** is adjusted P-value <0.01, * is adjusted P-value <0.05. N=3 independent experiments in all graphs. Source data are provided as a Source Data file.

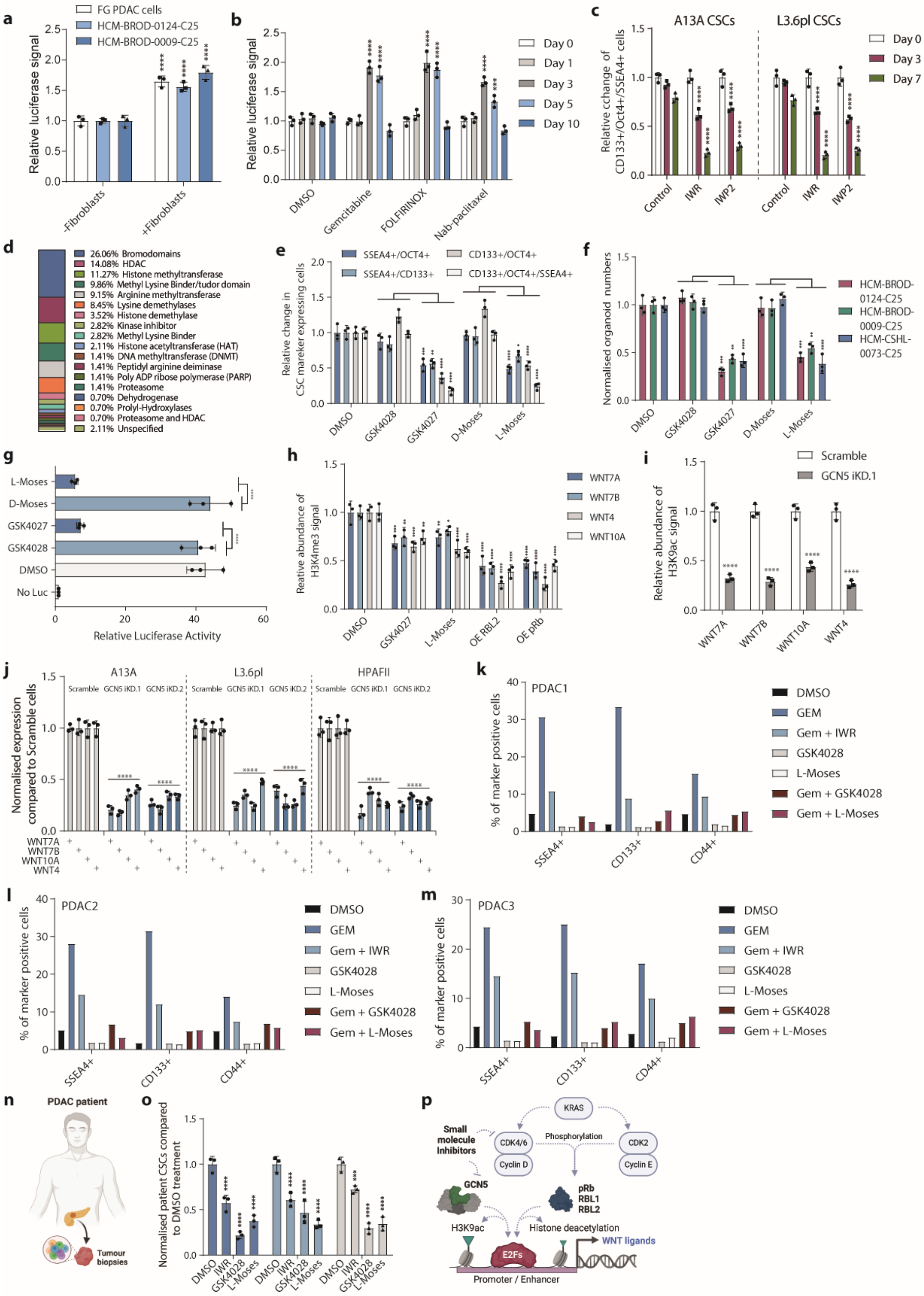

Supplementary Figure 8: Compounds screening identifies GCN5 as a regulator of CSCs that involves controlling WNT ligand expression. (**a**) Co-culturing of cancer cells with fibroblasts results in an increase in β-catenin dependent WNT signalling in cancer cells compared to culturing cancer cells without fibroblasts. (**b**) M50 Super 8x TOPFlash β-catenin-inducible promoter-luciferase assays indicate the dynamics of WNT/ β-catenin signalling upon chemotherapy treatment. (**c**) WNT signalling inhibition reduces CD133+/OCT4+/SSEA4+ CSCs in A13A and L3.6pl PDAC lines. (**d**) Small compound library constitution used for screening. (**e**) The reduction of CSC marker-expressing cells upon GCN5 inhibition. (**f**) GCN5 inhibition reduces organoid formation in PDAC patient-derived organoids. (**g**) GCN5 inhibition reduces β-catenin dependent transcription measured by promoter-luciferase assay. (**h**) OE pRB and OE RBL2 reduce H3K4me3 abundance on WNT ligand loci. Statistical analysis in g was performed by multiple t test. N=3 independent experiments. (**i**) GCN5 knockdown reduces H3K9ac abundance on WNT ligand loci. (**j**) GCN5 knockdown reduces WNT ligand expression in CSCs from three PDAC lines. (**k-m**) GCN5 inhibition reduces double and triple CSC marker positive CSCs from primary tumour sample from PDAC patients. (**n**) Schematic depiction of using primary tumour sample from PDAC patients. (**o**) The abundance of CSCs is decreased by GCN5 inhibitors in primary tumours from patients. (**p**) Schematic depiction of WNT ligand regulation by E2Fs, RBs and GCN5. Data are presented as mean values +/- SD. Statistical analysis in a, b, c, e, f, h, I, j, o was performed by 2-way ANOVA with multiple comparisons with Tukey correction and **** marks adjusted P-value <0.0001, *** is adjusted P-value <0.001, ** is adjusted P-value <0.01, * is adjusted P-value <0.05. N=3 independent experiments in all graphs. Source data are provided as a Source Data file.

Supplementary Figure 9: RBL2 is frequently mutated in a human primary breast cancer subgroup with poor clinical prognosis. (**a**) Correlation of E2Fs, CDKs and Cyclins with combined WNT ligands in normal mammary tissue. (**b**) Correlation of E2F1, E2F4 and CDK4 expression with WNT ligands in normal mammary tissue. (**c**) Correlation of E2F1 and E2F4 expression with WNT ligands in BRCA. (**d**) High expression of WNT7B, WNT3A and WNT10A correlates with lower survival of BRCA patients based on TCGA data. The stratified subtypes of BRCA are shown separately in addition

to the combined data for all BRCAs. (**e**) WNT7B, WNT3A and WNT9A expression in BRCA patients (T) compared to healthy controls (N). Statistical analysis is one-way ANOVA with p-value significance 0.01. The horizontal line shows mean value +/- SD. (**f**) RBL2 is frequently mutated in human primary breast tumors. Pie charts depicting the number of breast tumors with a CNA in pRb, RBL1 or RBL2 affecting expression, including the distribution of mutations in these loci. (**g**) Gene-centric frequencies of somatic mutations for RBL2 in human breast cancers according to PAM50 classification. (**h**) The clinical outcome of patients in 10 distinct breast cancer subgroups as described by [19]. Each of the ten breast cancer subgroups has also a distinct CNA classification and gene expression profile according to METABRIC database. (**i**) RB somatic mutation frequencies in the 10 primary breast cancer subgroups indicate frequent mutations of RBL2 in high mortality subcluster 2. (**j**) The high mortality breast cancer subgroup 2 has frequent somatic mutations in G1/S regulators and shows elevated expression of WNT pathway and stem cell like markers. (**k**) RBL2 and other G1/S regulators (Cyclin D, CDK4, E2F, pRb) are frequently mutated in poor-prognosis breast cancer subgroup 2 which also shows elevated expression of WNT pathway components. Green boxes highlight breast cancer subgroups with a higher frequency of mutations in the RBL2 locus, or the enrichment of mutations found in the G1/S regulator genes compared to other tumor subgroups. Red boxes highlight breast cancer subgroups with an elevated expression of WNT pathway components. (**l**) Comparison of RB protein expression in breast cancer lines. Western blot analysis of whole cells and the status of RBL2 in breast cancer lines used in experiments.

Supplementary Figure 10: RB pathway G1/S regulators exhibit autocrine effects on cancer cells through WNT ligands. (**a**) RBL2 overexpression decreases WNT ligand expression in BRCA cells. (**b**) RBL2 overexpression reduces β-catenin dependent transcription as analysed by promoter-luciferase assays. (**c**) Nuclear localisation of β-catenin in breast cancer cells is regulated by RBL2 and pRb via an autocrine mechanism. Immunostaining of β-catenin in HCC1500 breast cancer cells incubated for 12h with media from OE pRb cells or by adding canonical WNT inhibitor IWR. Scale bar 20µm. (**d**-**e**) RBs regulate WNT ligand expression in BRCA. (**d**) pRb overexpressed in breast cancer lines or (**e**) pRb or RBL1 were knocked down in HCC1500 breast cancer cells, and analysed by Q-PCR for WNT ligand expression. Significant differences compared to Scramble shRNA cells calculated by two-way ANOVA are marked. (**f-g**) Chemotherapy reagents enrich for BRCA cells expressing CSC markers. (f) HCC1500, CAL-51 and MDA-MB-231 cells were treated with 0.5µM Gemcitabine, 0.5µM FOLFIRINOX and 0.5µM Paclitaxel for 5 days and analysed by flow cytometry for detecting OCT4-GFP+/CD133+/SSEA4+ triple positive cells. (**g**) live cells upon each treatments. (**h**) GCN5 inhibition by small molecule inhibitors reduces the self-renewal of BRCA CSCs. Data are presented as mean values +/- SD. Statistical analysis in a, b, d, e, f, g, h was performed by 2-way ANOVA with multiple comparisons with Tukey correction and **** marks adjusted P-value <0.0001, *** is adjusted P-value <0.001, ** is adjusted P-value <0.01, * is adjusted P-value <0.05. N=3 independent experiments in all graphs. (**i**) GCN5 inhibition by small molecule inhibitors reduces β-catenin dependent transcription as analysed by promoter-luciferase assays. N=3 independent experiments in all graphs. Statistical analysis was performed by multiple t test. (**j**) Schematic overview of the autocrine effects of RBL2 on breast cancer stem cells in tumorigenic process. Source data are provided as a Source Data file.

Supplementary Figure 11: Schematic overview of the autocrine and paracrine effects of WNT ligands in CSCs. WNT ligands are regulated by pRb/RBL2-E2F1/4-GCN5 axis that controls cancer stem cell formation, chemoresistance, invasiveness and fibrosis.

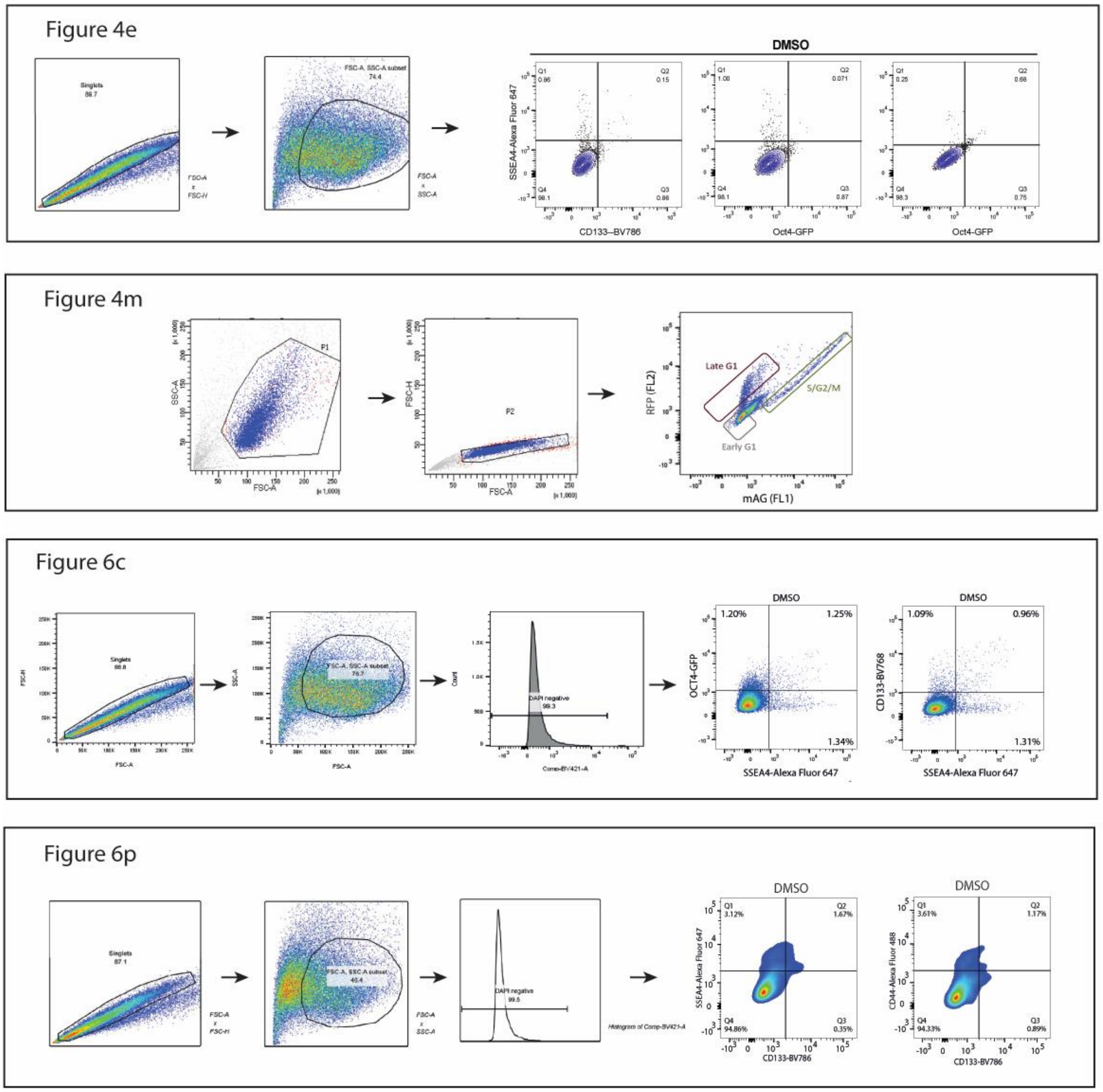

Supplementary Figure 12: Representative FACS sequential gating/sorting strategies.
