# Supplementary material for: The pRb/RBL2-E2F1/4-GCN5 axis regulates cancer stem cell formation and G0 phase entry/exit by paracrine mechanisms
Source: Nat Commun. 2024 Apr 27;15:3580. doi: 10.1038/s41467-024-47680-z (PMC11055877; doi:10.1038/s41467-024-47680-z)
Supplement: Supplementary file 2 — Reporting Summary [file 41467_2024_47680_MOESM2_ESM.pdf]

nature portfolio

Corresponding author(s): Siim Pauklin

Last updated by author(s): 06/04/2024

Reporting Summary

Nature Portfolio wishes to improve the reproducibility of the work that we publish. This form provides structure for consistency and transparency in reporting. For further information on Nature Portfolio policies, see our Editorial Policies and the Editorial Policy Checklist.

Statistics

For all statistical analyses, confirm that the following items are present in the figure legend, table legend, main text, or Methods section.

n/a	Confirmed	
☐	☒	The exact sample size (*n*) for each experimental group/condition, given as a discrete number and unit of measurement
☐	☒	A statement on whether measurements were taken from distinct samples or whether the same sample was measured repeatedly
☐	☒	The statistical test(s) used AND whether they are one- or two-sided<br>*Only common tests should be described solely by name; describe more complex techniques in the Methods section.*
☒	☐	A description of all covariates tested
☐	☒	A description of any assumptions or corrections, such as tests of normality and adjustment for multiple comparisons
☐	☒	A full description of the statistical parameters including central tendency (e.g. means) or other basic estimates (e.g. regression coefficient) AND variation (e.g. standard deviation) or associated estimates of uncertainty (e.g. confidence intervals)
☐	☒	For null hypothesis testing, the test statistic (e.g. *F*, *t*, *r*) with confidence intervals, effect sizes, degrees of freedom and *P* value noted<br>*Give P values as exact values whenever suitable.*
☒	☐	For Bayesian analysis, information on the choice of priors and Markov chain Monte Carlo settings
☒	☐	For hierarchical and complex designs, identification of the appropriate level for tests and full reporting of outcomes
☒	☐	Estimates of effect sizes (e.g. Cohen's *d*, Pearson's *r*), indicating how they were calculated

Our web collection on statistics for biologists contains articles on many of the points above.

Software and code

Policy information about availability of computer code

Data collection	- Flow cytometry data acquisition was performed using BD LSRFortessa cell analyzer (BD Biosciences) and collected with FACSDiva Software (BD, Version 8.0.1).<br>- Quantitative real-time PCR data were acquired using a ViiA7 Real-time PCR system (Applied Biosystems) with QuantStudio Software v1.6.1 (Applied Biosystems) and LightCycler 480 System (Roche).<br>- Tumorspheres data acquisition, including spheres count and imaging, was performed using Celigo Imaging Cytometer (Nexcelom Bioscience) with Celigo Software (Version 2.1).<br>- ChIP-seq and RNA-seq data were acquired using Illumina NovaSeq6000 S4. BCL convert v4.0.3 was used to generate the fastq raw data.<br>- A FluoStar Omega microplate reader (BMG Labtech) was used to measure the number of live cells stained with PrestoBlue cell viability reagent (Thermo Fisher Scientific, A13261) as per the manufacturer's instructions.
Data analysis	Publicly available softwares: FlowJo v10.8; Scaffold (version Scaffold_4.10.0); fastp v0.23.2; FastQC v0.11; Burrows-Wheeler Aligner v0.7.17; MACS2 v2.2.7.1; ChIPpeakAnno v3.30.0; DiffBind v3.6.1; STAR v2.7.3a; featureCounts v2.0.0; DESeq2 v1.34.0; fgsea v1.22.0; Molecular Signatures Database v7.5.1; g:GOSt; HDOCK; GraphPad Prism 8; and DNA Dynamo (Version 1.0).

For manuscripts utilizing custom algorithms or software that are central to the research but not yet described in published literature, software must be made available to editors and reviewers. We strongly encourage code deposition in a community repository (e.g. GitHub). See the Nature Portfolio guidelines for submitting code & software for further information.

Data

Policy information about availability of data

All manuscripts must include a data availability statement. This statement should provide the following information, where applicable:

- Accession codes, unique identifiers, or web links for publicly available datasets
- A description of any restrictions on data availability
- For clinical datasets or third party data, please ensure that the statement adheres to our policy

PDAC CSC ATAC-seq and RNA-seq data are available in the Gene Expression Ominbus (GEO) under accession code GSE244327 with no restrictions on data availability. The mass spectrometry proteomics data have been deposited to the ProteomeXchange Consortium via the PRIDE partner repository with the dataset identifier PXD038378 (Project Webpage: http://www.ebi.ac.uk/pride/archive/projects/PXD038378; FTP Download: ftp://ftp.pride.ebi.ac.uk/pride/data/archive/2023/08/PXD038378). The remaining data are available within the Article, Supplementary Information or Source Data file.

Human research participants

Policy information about studies involving human research participants and Sex and Gender in Research.

Reporting on sex and gender	The study did not involve human participants.
Population characteristics	The study did not involve human participants.
Recruitment	The study did not involve human participants.
Ethics oversight	The study did not involve human participants.

Note that full information on the approval of the study protocol must also be provided in the manuscript.

Field-specific reporting

Please select the one below that is the best fit for your research. If you are not sure, read the appropriate sections before making your selection.

- [x] Life sciences
- [ ] Behavioural & social sciences
- [ ] Ecological, evolutionary & environmental sciences

For a reference copy of the document with all sections, see nature.com/documents/nr-reporting-summary-flat.pdf

Life sciences study design

All studies must disclose on these points even when the disclosure is negative.

Sample size	No statistical methods were used to predetermine the sample size for in vitro and in vivo experiments, but preliminary experiments were conducted to determine the appropriate sample size that would provide sufficient statistical power. For in vitro experiments, 3-6 biological replicates were used, which allowed us to perform statistical analysis.
Data exclusions	No data were excluded from the study.
Replication	3-6 independent sample replicates were used for each experiment as indicated in the figure legends. All attempts at replication were successful.
Randomization	- For in vitro functional assays and RNA-seq analysis, pancreatic cancer stem cells were randomized for vehicle and drug treatments.<br>- For all other in vitro experiments including mass spectrometry, RT-qPCR, Co-IP and western blotting, histone peptides pull down assay, ChIP-seq, and small molecule compound screening, samples were randomized into the indicated groups.
Blinding	- Mass Spectrometry analysis was performed at the Proteomics Facility at Target Discovery Institute (University of Oxford) where codes have been used to identify samples. Data were acquired by one researcher at the facility and analyzed by a different researcher in a blinded manner.<br>- ATAC-seq and RNA-seq data acquisition was carried out at University of Oxford, using sample codes to identify submitted samples, and data were analyzed by an independent bioinformatician.<br>- Blinding was not possible for all other data based on quantitative analysis during the conductance of the experiments for appropriate treatments to the assigned groups. However, the investigators and authors have been consistently blinded to the group allocation during data collection and analysis

Reporting for specific materials, systems and methods

We require information from authors about some types of materials, experimental systems and methods used in many studies. Here, indicate whether each material, system or method listed is relevant to your study. If you are not sure if a list item applies to your research, read the appropriate section before selecting a response.

Materials & experimental systems

n/a	Involved in the study
☐	☒ Antibodies
☐	☒ Eukaryotic cell lines
☒	☐ Palaeontology and archaeology
☒	☐ Animals and other organisms
☒	☐ Clinical data
☒	☐ Dual use research of concern

Methods

n/a	Involved in the study
☐	☒ ChIP-seq
☐	☒ Flow cytometry
☒	☐ MRI-based neuroimaging

Antibodies

Antibodies used

For Flow Cytometry: PerCP/Cy5.5 anti-ABCG2 (BioLegend, 332024; 1:50), APC anti-EPCAM (BioLegend, 324208; 1:50), PE anti-CD44 (BD Biosciences, 555479; 1:10), BV421 anti-CD24 (BD Biosciences, 562789; 1:50), BV786 anti-PROM1 (BD Biosciences, 747640; 1:50), FITC anti-SSEA4 (BD Biosciences, 560126; 1:10),BV510 anti-CXCR4 (BioLegend, 306535; 1:50), PerCP-Cy5.5 mouse IgG2b, k (BD Biosciences, 558020; 1:50), APC mouse IgG2b, k (BioLegend, 402206; 1:50), PE mouse IgG2a, k (BD Biosciences, 555574; 1:10), BV421 mouse IgG2a, k (BD Biosciences, 562439; 1:50), BV786 mouse IgG1, k (BD Biosciences, 563330; 1:50), FITC mouse IgG3, k (BD Biosciences, 556658; 1:10), and BV510 mouse IgG2a, k (BioLegend, 400267; 1:50), Pacific Blue anti-SOX2 antibody (BioLegend, 656111; 1:20), Alexa Fluor 647 anti-SSEA4 (BD Biosciences, 560796; 1:40) and its isotype control Alexa Fluor 647 Mouse IgG3, k (BD Biosciences, 560803; 1:40).
For MACS: Magnetic microbeads conjugated with anti-ABCG2 (Miltenyi Biotec, 130-107-680; 1:5) and anti-SSEA4 (Miltenyi Biotec, 130-097-855; 1:5).
For Co-immunoprecipitation: anti-SMAD2/3 (R and D Systems, AF3797; 1:200), normal goat IgG (R and D Systems, AB-108-C; 1:200), and rabbit (DA1E) mAb IgG XP (Cell Signaling Technology, 3900S; 1:500).
For Western blotting: SMAD2/3 (Cell Signaling Technology, 3102S; 1:2000), p-SMAD2 (Ser465/Ser467) (Thermo Fisher Scientific, 44-244G; 1:1000), p-SMAD3 (Ser423/Ser425) (Abcam, ab52903; 1:2000), SMAD4 (Proteintech, 10231-1-AP; 1:2000), α-Tubulin (Proteintech, 66031-1-Ig; 1:6000), Lamin B1 (Proteintech, 12987-1-AP; 1:2000), H3K4me3 (Abcam, ab213224; 1:2000), Histone H3 (Abcam, ab1791; 1:6000), anti-rabbit (Sigma-Aldrich, A0545; 1:10,000) or anti-mouse (Sigma-Aldrich, A9044; 1:10,000) horseradish peroxidase (HRP)-conjugated secondary antibodies.

Validation

All antibodies were commercially validated (see manufacturer's website link below), previously published or validated in our study.
Pacific Blue anti-SOX2 (BioLegend, cat. 656111), https://www.biolegend.com/en-gb/search-results/pacific-blue-anti-sox2-antibody-12471?GroupID=GROUP26.
Anti-SMAD2/3 (Cell Signaling Technology, cat. 3102S), https://www.cellsignal.com/products/primary-antibodies/smad2-3-antibody/3102
Anti-phospho-SMAD2 (Ser465/Ser467) (Thermo Fisher Scientific, cat. 44-244G), https://www.thermofisher.com/antibody/product/Phospho-SMAD2-Ser465-Ser467-Antibody-Polyclonal/44-244G
Anti-phospho-SMAD3 (Ser423/Ser425) (Abcam, ab52903), https://www.thermofisher.com/antibody/product/Phospho-SMAD3-Ser423-Ser425-Antibody-Polyclonal/44-246G.
Anti-SMAD4 (Proteintech, 10231-1-AP), https://www.ptglab.com/products/SMAD4-Antibody-10231-1-AP.htm.
Anti-alpha Tubulin (Proteintech, cat. 66031-1-Ig), https://www.ptglab.com/products/tubulin-Alpha-Antibody-66031-1-Ig.htm
Anti-H3K4me3 (Abcam, cat. ab213224), https://www.abcam.com/histone-h3-tri-methyl-k4-antibody-epr20551-225-chip-grade-ab213224.html
Anti-Histone H3 (Abcam, cat. ab1791), https://www.abcam.com/histone-h3-antibody-nuclear-marker-and-chip-grade-ab1791.html
Anti-rabbit HRP-conjugated secondary antibody (Sigma-Aldrich, cat. A0545) , https://www.sigmaaldrich.com/EG/en/product/sigma/a0545
Anti-mouse HRP-conjugated secondary antibody (Sigma-Aldrich, cat. A9044), https://www.sigmaaldrich.com/EG/en/product/sigma/a9044)
PerCP/Cy5.5 anti-ABCG2 (BioLegend, cat. 332024), https://www.biolegend.com/en-us/products/percp-cyanine5-5-anti-human-cd20-antibody-4228?GroupID=BLG7904
APC anti-EPCAM (BioLegend, cat. 324208), https://www.biolegend.com/en-us/products/apc-anti-mouse-cd326-ep-cam-antibody-4974?GroupID=BLG5748
PE anti-CD44 (BD Biosciences, cat. 555479), https://www.bdbiosciences.com/en-us/products/reagents/flow-cytometry-reagents/research-reagents/single-color-antibodies-ruo/pe-mouse-anti-human-cd44.555479
BV421 anti-CD24 (BD Biosciences, cat. 562789), https://www.bdbiosciences.com/en-us/products/reagents/flow-cytometry-reagents/research-reagents/single-color-antibodies-ruo/bv421-mouse-anti-human-cd24.562789
BV786 anti-PROM1 (BD Biosciences, cat. 747640), https://www.bdbiosciences.com/en-us/products/reagents/flow-cytometry-reagents/research-reagents/single-color-antibodies-ruo/bv786-mouse-anti-human-cd133.747640
FITC anti-SSEA4 (BD Biosciences, cat. 560126), https://www.bdbiosciences.com/en-us/products/reagents/flow-cytometry-reagents/research-reagents/single-color-antibodies-ruo/fitc-mouse-anti-ssea-4.560126
BV510 anti-CXCR4 (BioLegend, cat. 306535), https://punchout.biolegend.com/en-us/search-results/brilliant-violet-510-anti-human-cd184-cxcr4-antibody-17653?GroupID=BLG8071
PerCP-Cy5.5 mouse IgG2b, k (BD Biosciences, cat. 558020), https://www.bdbiosciences.com/en-us/products/reagents/flow-cytometry-reagents/research-reagents/flow-cytometry-controls-and-lysates/percp-cy-5-5-mouse-igg2a-isotype-control.558020
APC mouse IgG2b, k (BioLegend, cat. 402206), https://www.biolegend.com/en-us/products/apc-mouse-igg2b-kappa-isotype-ctrl-14793

PE mouse IgG2a, k (BD Biosciences, cat. 555574), https://www.bdbiosciences.com/en-us/products/reagents/flow-cytometry-reagents/research-reagents/flow-cytometry-controls-and-lysates/pe-mouse-igg2a-isotype-control.555574
BV421 mouse IgG2a, k (BD Biosciences, cat. 562439), https://www.bdbiosciences.com/en-us/products/reagents/flow-cytometry-reagents/research-reagents/flow-cytometry-controls-and-lysates/bv421-mouse-igg2a-k-isotype-control.562439
BV786 mouse IgG1, k (BD Biosciences, cat. 563330), https://www.bdbiosciences.com/en-us/products/reagents/flow-cytometry-reagents/research-reagents/flow-cytometry-controls-and-lysates/bv786-mouse-igg1-k-isotype-control.563330
FITC mouse IgG3, k (BD Biosciences, cat. 556658), https://www.bdbiosciences.com/en-us/products/reagents/flow-cytometry-reagents/research-reagents/flow-cytometry-controls-and-lysates/fitc-mouse-igg3-isotype-control.556658
BV510 mouse IgG2a, k (BioLegend, cat. 400267), https://www.biolegend.com/en-us/products/brilliant-violet-510-mouse-igg2a-kappa-isotype-ctrl-8015

Eukaryotic cell lines

Policy information about cell lines and Sex and Gender in Research

Cell line source(s)

L3.6pl, L3.6sl, and FG pancreatic cancer cell lines were purchased from MD Anderson Cancer Center (USA). A13A and A13D pancreatic cancer cell lines were kindly gifted by Professor Christine Iacobuzio-Donahue (Memorial Sloan Kettering Cancer Center, USA) in 2018 and 2020, respectively. The immortalized human pancreatic ductal epithelial cell line HPDE6c7 was purchased from Kerafast, cat. no ECA001-FP (USA).

Authentication

L3.6pl, L3.6sl, FG, HPAFII, A13A, and HPDE6c7 cell lines were authenticated using Short Tandem Repeat DNA profiling before we obtained them. Furthermore, cell lines were routinely authenticated and checked for mycoplasma infection.

Mycoplasma contamination

A PCR-based method for the detection of Mycoplasma contamination in cell cultures was routinely performed using the following primer sequences: forward primer; 5’-GGGAGCAAACAGGATTAGATACCCT-3’ and reverse primer; 5’-TGCACCATCTGTCACTCTGTTAACCTC-3’. All cell lines tested negative for mycoplasma contamination.

Commonly misidentified lines
(See ICLAC register)

No commonly misidentified cell lines were used in this study.

ChIP-seq

Data deposition

☒ Confirm that both raw and final processed data have been deposited in a public database such as GEO.

☒ Confirm that you have deposited or provided access to graph files (e.g. BED files) for the called peaks.

Data access links
May remain private before publication.

PDAC CSC ATAC-seq are available in the Gene Expression Ominbus (GEO) under accession code GSE244327 with no restrictions on data availability.

Files in database submission

ATAC-seq data:
*processed data file
A13A_Adh_Ctrl_Rep_1.peaks
A13A_Adh_Ctrl_Rep_2.peaks
A13A_Adh_Ctrl_Rep_3.peaks
A13A_P_Ctrl_Rep_1.peaks
A13A_P_Ctrl_Rep_2.peaks
A13A_P_Ctrl_Rep_3.peaks

*raw file
i16-A13A-Adh-Ctrl-Rep-I_S21_L001_R1_001.fastq
i17-A13A-Adh-Ctrl-Rep-II_S5_L001_R1_001.fastq
i18-A13A-Adh-Ctrl-Rep-III_S8_L001_R1_001.fastq
i7-A13A-P-Ctrl-Rep-I_S17_L001_R1_001.fastq
i8-A13A-P-Ctrl-Rep-II_S9_L001_R1_001.fastq
i9-A13A-P-Ctrl-Rep-III_S2_L001_R1_001.fastq

raw file
i16-A13A-Adh-Ctrl-Rep-I_S21_L001_R2_001.fastq
i17-A13A-Adh-Ctrl-Rep-II_S5_L001_R2_001.fastq
i18-A13A-Adh-Ctrl-Rep-III_S8_L001_R2_001.fastq
i7-A13A-P-Ctrl-Rep-I_S17_L001_R2_001.fastq
i8-A13A-P-Ctrl-Rep-II_S9_L001_R2_001.fastq
i9-A13A-P-Ctrl-Rep-III_S2_L001_R2_001.fastq

raw file
i16-A13A-Adh-Ctrl-Rep-I_S21_L002_R1_001.fastq
i17-A13A-Adh-Ctrl-Rep-II_S5_L002_R1_001.fastq
i18-A13A-Adh-Ctrl-Rep-III_S8_L002_R1_001.fastq

i7-A13A-P-Ctrl-Rep-I_S17_L002_R1_001.fastq
i8-A13A-P-Ctrl-Rep-II_S9_L002_R1_001.fastq
i9-A13A-P-Ctrl-Rep-III_S2_L002_R1_001.fastq

raw file
i16-A13A-Adh-Ctrl-Rep-I_S21_L002_R2_001.fastq
i17-A13A-Adh-Ctrl-Rep-II_S5_L002_R2_001.fastq
i18-A13A-Adh-Ctrl-Rep-III_S8_L002_R2_001.fastq
i7-A13A-P-Ctrl-Rep-I_S17_L002_R2_001.fastq
i8-A13A-P-Ctrl-Rep-II_S9_L002_R2_001.fastq
i9-A13A-P-Ctrl-Rep-III_S2_L002_R2_001.fastq

*raw file
i16-A13A-Adh-Ctrl-Rep-I_S21_L003_R1_001.fastq
i17-A13A-Adh-Ctrl-Rep-II_S5_L003_R1_001.fastq
i18-A13A-Adh-Ctrl-Rep-III_S8_L003_R1_001.fastq
i7-A13A-P-Ctrl-Rep-I_S17_L003_R1_001.fastq
i8-A13A-P-Ctrl-Rep-II_S9_L003_R1_001.fastq
i9-A13A-P-Ctrl-Rep-III_S2_L003_R1_001.fastq

raw file
i16-A13A-Adh-Ctrl-Rep-I_S21_L003_R2_001.fastq
i17-A13A-Adh-Ctrl-Rep-II_S5_L003_R2_001.fastq
i18-A13A-Adh-Ctrl-Rep-III_S8_L003_R2_001.fastq
i7-A13A-P-Ctrl-Rep-I_S17_L003_R2_001.fastq
i8-A13A-P-Ctrl-Rep-II_S9_L003_R2_001.fastq
i9-A13A-P-Ctrl-Rep-III_S2_L003_R2_001.fastq

raw file
i16-A13A-Adh-Ctrl-Rep-I_S21_L004_R2_001.fastq
i17-A13A-Adh-Ctrl-Rep-II_S5_L004_R2_001.fastq
i18-A13A-Adh-Ctrl-Rep-III_S8_L004_R2_001.fastq
i7-A13A-P-Ctrl-Rep-I_S17_L004_R2_001.fastq
i8-A13A-P-Ctrl-Rep-II_S9_L004_R2_001.fastq
i9-A13A-P-Ctrl-Rep-III_S2_L004_R2_001.fastq

RNA-seq data:
*processed data file
A13A_RNAseq_Count table

*raw file
i19-A13A-Adh-Ctrl-Rep1_S8_L001_R1_001.fastq
i20-A13A-Adh-Ctrl-Rep2_S23_L002_R1_001.fastq
i21-A13A-Adh-Ctrl-Rep3_S19_L001_R1_001.fastq
i46-A13A-P-Ctrl-Rep1_S23_L001_R1_001.fastq
i47-A13A-P-Ctrl-Rep2_S20_L001_R1_001.fastq
i48-A13A-P-Ctrl-Rep3_S3_L001_R1_001.fastq

raw file
i19-A13A-Adh-Ctrl-Rep1_S8_L001_R2_001.fastq
i20-A13A-Adh-Ctrl-Rep2_S23_L002_R2_001.fastq
i21-A13A-Adh-Ctrl-Rep3_S19_L001_R2_001.fastq
i46-A13A-P-Ctrl-Rep1_S23_L001_R2_001.fastq
i47-A13A-P-Ctrl-Rep2_S20_L001_R2_001.fastq
i48-A13A-P-Ctrl-Rep3_S3_L001_R2_001.fastq

raw file
i19-A13A-Adh-Ctrl-Rep1_S8_L002_R1_001.fastq
i20-A13A-Adh-Ctrl-Rep2_S23_L003_R1_001.fastq
i21-A13A-Adh-Ctrl-Rep3_S19_L002_R1_001.fastq
i46-A13A-P-Ctrl-Rep1_S23_L002_R1_001.fastq
i47-A13A-P-Ctrl-Rep2_S20_L002_R1_001.fastq
i48-A13A-P-Ctrl-Rep3_S3_L002_R1_001.fastq

raw file
i19-A13A-Adh-Ctrl-Rep1_S8_L002_R2_001.fastq
i20-A13A-Adh-Ctrl-Rep2_S23_L003_R2_001.fastq
i21-A13A-Adh-Ctrl-Rep3_S19_L002_R2_001.fastq

i46-A13A-P-Ctrl-Rep1_S23_L002_R2_001.fastq
i47-A13A-P-Ctrl-Rep2_S20_L002_R2_001.fastq
i48-A13A-P-Ctrl-Rep3_S3_L002_R2_001.fastq

*raw file
i19-A13A-Adh-Ctrl-Rep1_S8_L003_R1_001.fastq
i20-A13A-Adh-Ctrl-Rep2_S23_L003_R1_001.fastq
i21-A13A-Adh-Ctrl-Rep3_S19_L003_R1_001.fastq
i46-A13A-P-Ctrl-Rep1_S23_L003_R1_001.fastq
i47-A13A-P-Ctrl-Rep2_S20_L003_R1_001.fastq
i48-A13A-P-Ctrl-Rep3_S3_L003_R1_001.fastq

raw file
i19-A13A-Adh-Ctrl-Rep1_S8_L003_R2_001.fastq
i20-A13A-Adh-Ctrl-Rep2_S23_L003_R2_001.fastq
i21-A13A-Adh-Ctrl-Rep3_S19_L003_R2_001.fastq
i46-A13A-P-Ctrl-Rep1_S23_L003_R2_001.fastq
i47-A13A-P-Ctrl-Rep2_S20_L003_R2_001.fastq
i48-A13A-P-Ctrl-Rep3_S3_L003_R2_001.fastq

raw file
i19-A13A-Adh-Ctrl-Rep1_S8_L004_R1_001.fastq
i20-A13A-Adh-Ctrl-Rep2_S23_L004_R1_001.fastq
i21-A13A-Adh-Ctrl-Rep3_S19_L004_R1_001.fastq
i46-A13A-P-Ctrl-Rep1_S23_L004_R1_001.fastq
i47-A13A-P-Ctrl-Rep2_S20_L004_R1_001.fastq
i48-A13A-P-Ctrl-Rep3_S3_L004_R1_001.fastq

raw file
i19-A13A-Adh-Ctrl-Rep1_S8_L004_R2_001.fastq
i20-A13A-Adh-Ctrl-Rep2_S23_L004_R2_001.fastq
i21-A13A-Adh-Ctrl-Rep3_S19_L004_R2_001.fastq
i46-A13A-P-Ctrl-Rep1_S23_L004_R2_001.fastq
i47-A13A-P-Ctrl-Rep2_S20_L004_R2_001.fastq
i48-A13A-P-Ctrl-Rep3_S3_L004_R2_001.fastq

Genome browser session
(e.g. UCSC)

Provide a link to an anonymized genome browser session for "Initial submission" and "Revised version" documents only, to enable peer review. Write "no longer applicable" for "Final submission" documents.

Methodology

Replicates

3 biological replicates per each experiment.

Sequencing depth

Describe the sequencing depth for each experiment, providing the total number of reads, uniquely mapped reads, length of reads and whether they were paired- or single-end.

Antibodies

N/A

Peak calling parameters

Peaks were called using MACS2 v2.2.7.1.

Data quality

Raw reads were cleaned using fastp v0.23.2 with default parameters. Cleaned reads were confirmed high-quality using FastQC v0.11.9. Duplicated reads, reads mapped to ENCODE blacklisted regions , and reads with mapping quality lower than 30 were removed, and only properly paired reads were retained.

Software

BCL convert v4.0.3 was used to generate the fastq raw data. Raw reads were cleaned using fastp v0.23.2 with default parameters. Cleaned reads were then confirmed high-quality using FastQC v0.11. Burrows-Wheeler Aligner v0.7.17 was used to map cleaned reads to the human genome hg38. Duplicated reads, reads mapped to ENCODE blacklisted regions and reads with a mapping quality lower than 30 were removed, and only properly paired reads were retained. Peaks were then called using MACS2 v2.2.7.1.

Flow Cytometry

Plots

Confirm that:

- [x] The axis labels state the marker and fluorochrome used (e.g. CD4-FITC).
- [x] The axis scales are clearly visible. Include numbers along axes only for bottom left plot of group (a 'group' is an analysis of identical markers).
- [x] All plots are contour plots with outliers or pseudocolor plots.
- [x] A numerical value for number of cells or percentage (with statistics) is provided.

Methodology

Sample preparation	- Flow Cytometry analysis of enriched CSC surface markers in pancreatic tumorspheres: Single cells were suspended in ice-cold cell staining buffer consisting of DPBS (Thermo Fisher Scientific, cat. 14190169), 10% heat-inactivated FBS (Sigma-Aldrich, cat. F9665) and 0.1% sodium azide (Sigma-Aldrich, cat. S2002) then incubated with the Fc receptor blocking reagent human TrueStain FcX (Biolegend, cat. 422301) for 10 minutes at room temperature. Cells were labeled with fluorochrome-conjugated antibodies. Following labeling, cells were washed 3 times with the cell staining buffer then incubated with DAPI (BD Biosciences, cat. 564907) at a final concentration of 0.1 µg/ml in cell staining buffer for 15 minutes at room temperature in the dark for assessment of cell viability. Fluorescence minus one (FMO) and isotype control antibodies were used to gate and identify the positive cell population.
Instrument	Flow cytometry data were acquired using BD LSRFortessa cell analyzer (BD Biosciences).
Software	Flow cytometry data were collected with FACSDiva Software (BD , Version 8.0.1) and analyzed using FlowJo v10.8 Software (BD Life Sciences).
Cell population abundance	At passages 3-4, enrichment of pancreatic cancer stem cells in tumorspheres was tested by flow cytometry, where only batches showing at least 85% of CSC marker enrichment were used for subsequent experiments.
Gating strategy	FSC-A/SSC-A gates were used to eliminate cell debris from analysis > FSC-H/SSC-H gates were used to identify single cells > FSC-A/DAPI gates were used to eliminate dead (DAPI+) cells from the analysis. Both isotype and FMO controls were used to gate the positive cell population.

- [x] Tick this box to confirm that a figure exemplifying the gating strategy is provided in the Supplementary Information.
