# Supplementary material for: The pRb/RBL2-E2F1/4-GCN5 axis regulates cancer stem cell formation and G0 phase entry/exit by paracrine mechanisms
Source: Nat Commun. 2024 Apr 27;15:3580. doi: 10.1038/s41467-024-47680-z (PMC11055877; doi:10.1038/s41467-024-47680-z)
Supplement: Supplementary file 3 — Description of Additional Supplementary Files [file 41467_2024_47680_MOESM3_ESM.pdf]

Description of Additional Supplementary Files

Supplementary Data 1 - Small molecule compounds used in the screening experiment. List with compound names, working concentrations and target molecules.
